# Body shape differences in a pair of closely related Malawi cichlids and their hybrids: Effects of genetic variation, phenotypic plasticity, and transgressive segregation

**DOI:** 10.1002/ece3.2823

**Published:** 2017-05-10

**Authors:** Martin Husemann, Michael Tobler, Cagney McCauley, Baoqing Ding, Patrick D. Danley

**Affiliations:** ^1^ Centrum für NaturkundeUniversity of HamburgHamburgGermany; ^2^ Biology DepartmentBaylor UniversityWacoTXUSA; ^3^ Division of BiologyKansas State UniversityManhattanKSUSA; ^4^ Department of Biological SciencesInstitute of Applied SciencesUniversity of North Texas282 Cr 332 RosebudDentonTXUSA; ^5^ Department of Ecology and Evolutionary BiologyUniversity of ConnecticutStorrsCTUSA

**Keywords:** cichlids, geometric morphometrics, Lake Malawi, local adaptation, phenotypic plasticity, transgressive segregation

## Abstract

Phenotypic differences may have genetic and plastic components. Here, we investigated the contributions of both for differences in body shape in two species of Lake Malawi cichlids using wild‐caught specimens and a common garden experiment. We further hybridized the two species to investigate the mode of gene action influencing body shape differences and to examine the potential for transgressive segregation. We found that body shape differences between the two species observed in the field are maintained after more than 10 generations in a standardized environment. Nonetheless, both species experienced similar changes in the laboratory environment. Our hybrid cross experiment confirmed that substantial variation in body shape appears to be genetically determined. The data further suggest that the underlying mode of gene action is complex and cannot be explained by simple additive or additive‐dominance models. Transgressive phenotypes were found in the hybrid generations, as hybrids occupied significantly more morphospace than both parentals combined. Further, the body shapes of transgressive individuals resemble the body shapes observed in other Lake Malawi rock‐dwelling genera. Our findings indicate that body shape can respond to selection immediately, through plasticity, and over longer timescales through adaptation. In addition, our results suggest that hybridization may have played an important role in the diversification of Lake Malawi cichlids through creating new phenotypic variation.

## 1 Introduction

Understanding the drivers of phenotypic diversification remains one of the central goals of evolutionary biology. Recent, rapid radiations represent optimal systems to study the forces underlying diversification, as they are characterized by large amounts of phenotypic variation with a common origin and often a young phylogenetic age. Multiple underlying factors may contribute to phenotypic divergence, and dissecting the different components may not always be easy. Historically, mutation and recombination were believed to be the primary source of phenotypic variation for selection to act upon. However, additional mechanisms have been recognized as generators of phenotypic variation.

Two mechanisms in particular, phenotypic plasticity and transgressive segregation, can provide phenotypic variation during rapid radiations. Phenotypic plasticity may generate new phenotypes selection can act upon (Moser, Kueng, & Berner, 2015; Pfennig et al., 2010). Phenotypic plasticity is determined by the intraspecific genetic architecture and refers to the effect the environment has on phenotypic expression (Schreiner, 1993). The different phenotypes a genotype may express are defined in the reaction norm. Likewise, transgressive segregation can generate the phenotypic variation (Rieseberg, Archer, & Wayne, 1999; Seehausen, 2004) that seeds adaptive radiations (Selz, Lucek, Young, & Seehausen, 2014). Transgressive segregation occurs when hybrid phenotypes exceed the phenotypic distribution of the parental species (Rieseberg et al., 1999). Individuals possessing these novel phenotypes may then occupy novel niches and gain a selective advantage (Ghalambor, McKay, Carroll, & Reznik, 2007; Seehausen, 2004). Transgressive segregation is expected to increase with increasing genetic distance between the parental species (Stelkens, Schmid, Selz, & Seehausen, 2009; Stelkens & Seehausen, 2009) due to complementary gene action with antagonistic effects (Rieseberg et al., 1999). Therefore, recently separated species driven apart by consistent divergent selection are not expected to exhibit transgressive segregation (Albertson & Kocher, 2005). However, due to their potential to generate phenotypic variation, both phenotypic plasticity and transgressive segregation have been suggested to play crucial roles in adaptive radiations (Genner & Turner, 2012; Seehausen, 2004; Selz et al., 2014).

One of the most diverse and best‐known adaptive radiation is represented by the East African cichlid fish. More than 2000 species of haplochomine cichlids in the three East African Great Lakes (Tanganyika, Victoria, and Malawi) exhibit an extraordinary amount of phenotypic diversity allowing cichlids to occupy all major ecological niches within the lakes (Seehausen, 2006; Sturmbauer, Husemann, & Danley, 2011). Of the three lakes, Lake Malawi harbors the most species‐rich radiation with more than 700 species (Danley et al., 2012). Selection is thought to be the main driver of diversification in the different stages of the cichlid radiations (Danley & Kocher, 2001; Muschick et al., 2014). In the early stages, natural selection may lead to macrohabitat divergence and the differentiation of genera with different trophic traits, respectively. The divergence in trophic traits can be accompanied by the divergence in body shapes helping to exploit a variety of resources and microhabitats (Hulsey, Roberts, Loh, Rupp, & Streelman, 2013; Husemann, Tobler, McCauley, Ding, & Danley, 2014) leading to highly complex communities (Ding, Daugherty et al., 2014). During the most recent stage of diversification, natural and sexual selection drove the divergence of signaling phenotypes, microhabitat preferences, and body morphologies (Husemann et al., 2014; Kerschbaumer, Mitteroecker, & Sturmbauer, 2013; Streelman, Albertson, & Kocher, 2007; Sturmbauer, 1998). Hence, body shape is a key trait at multiple levels of the cichlid radiation.

Although body shape in fish can evolve in response to a variety of evolutionary forces, including predation (Langerhans, 2009), abiotic environmental factors (Neves & Monteiro, 2003), and competition (Scott & Johnson, 2010), it often evolves in response to ecological selection and can be used as an ecological marker when studying differentiation in natural populations (Tobler et al., 2008). A classic axis of body shape divergence in fishes is the divergence into deep‐bodied and slender‐bodied morphs associated with adaptation to benthic and limnetic macrohabitats. This pattern has been documented in a variety of species (e.g., Schluter, 1993; Willacker, Hippel, Wilton, & Walton, 2010) including Lake Malawi cichlids (Hulsey et al., 2013).

With 31 described species, *Maylandia* is among the most species‐rich genera in Lake Malawi (Stauffer, Black, & Konings, 2013). Species in this genus exhibit inter‐ and intraspecific differences in male mating coloration, body shape, and behavior (Danley, 2011; Husemann et al., 2014; Kidd, Danley, & Kocher, 2006; Stauffer et al., 2013). However, the underlying mechanisms which drove this divergence remain unknown. While several studies have addressed the genetic makeup, plasticity, and transgression of body shape in other cichlid radiations (e.g., Franchini et al., 2014; Kerschbaumer, Postl, Koch, Wiedl, & Sturmbauer, 2011; Kerschbaumer et al., 2013; Klingenberg, Barluenga, & Meyer, 2003; Selz et al., 2014; Stelkens et al., 2009), we have relatively little information on Lake Malawi cichlids. To gain a better understanding of the forces driving body shape evolution in Malawi cichlids, we performed a common garden experiment and generated hybrid crosses between two closely related species of the genus *Maylandia*. We expected the main differences between species remaining stable in fish bred under standardized conditions, yet also predicted a plastic response to the new environment. Our experimental design further allowed us to investigate the mode of gene action underlying the differences in body shape and to determine the potential for transgressive segregation. As body shape is a complex, modular phenotype, we expected that the differences would not be explained by a simple additive model, but rather involve epistatic interactions, as it has been shown for example in sticklebacks (Schluter, Clifford, Nemethy, & McKinnon, 2004). Complex gene interactions are thought to promote transgressive segregation, generating new body shape phenotypes as a result of hybridization (Rieseberg, Widmer, Arntz, & Burke, 2003; Rieseberg et al., 1999; Tobler & Carson, 2010). However, we expected transgression to be limited in this pair of closely related species, as the amount of transgression exhibited in a cross is often correlated with the genetic distance between the parentals (Stelkens & Seehausen, 2009; Stelkens et al., 2009). Overall, we anticipated to gain an insight into the roles of plasticity and transgression for body shape divergence in the adaptive radiation of Lake Malawi cichlids.

## 2 Material and Methods

### 2.1 Study species

We used two closely related species of the rock‐dwelling cichlid genus *Maylandia* to study the environmental and genetic components of body shape differentiation and to test for transgressive segregation. *Maylandia benetos* is a microendemic only occurring at a single location in the lake, Mazinzi Reef, where it is sympatric with three other *Maylandia* species, including *Maylandia zebra*. *Maylandia zebra* is one of the few rock‐dwelling cichlids (mbuna) found at most rocky habitats throughout the lake (Ribbink, Marsh, Marsh, Ribbink, & Sharp, 1983). Both species can be readily distinguished by their body coloration, distinct behavior, and microhabitat choice (Danley, 2011; Ding, Curole, Husemann, & Danley, 2014; Husemann et al., 2014). The species do not hybridize in nature, but can produce viable and fertile offspring if artificially fertilized (Ding, Daugherty et al., 2014). A previous study has shown that sympatric barred and nonbarred *Maylandia* species, including *M. benetos* (nonbarred) and *M. zebra* (barred) from Mazinzi Reef, are differentiated in their body shape in a predictable manner (Husemann et al., 2014). To further understand the repeated, parallel divergence of body shape differentiating barred and nonbarred species, we used *M. benetos* and *M. zebra* as a model to study the transmission of body shape variation and to understand potential mechanisms creating phenotypic divergence in Lake Malawi cichlids. Specifically, (1) populations of both species were raised in identical aquaculture environments, resembling neither of the natural niches of either species, to quantify the degree of plasticity influencing this phenotype, and (2) these species were artificially hybridized in the laboratory to investigate the underlying mode of gene action and the amount of transgressive segregation observed in the body shape phenotype.

### 2.2 Field sampling and laboratory breeding conditions

We collected adult specimens of *M. zebra* (*N* = 38) and *M. benetos* (*N* = 44) in the summers of 2010 and 2012 at Mazinzi Reef. Specimens were caught in nets while using SCUBA and photographed using a Canon Eos 540d. In addition, we analyzed *M. zebra* (*N* = 81) and *M. benetos* (*N* = 55) descended from wild‐caught populations that have been maintained as laboratory stocks for approximately 12 generations. Fish were kept in 110 cm × 28 cm × 30 cm tanks at water temperatures between 26 and 28°C. Light was kept at a 12‐hr day/night cycle using timer‐controlled fluorescent lights. Fish were fed a mixture of food flakes twice daily.

Bidirectional F_1_ (*N* = 96) were produced from five independent broods, and these F_1_ were used to produce F_2_ (*N* = 326) from three independent crosses. We then generated one backcross with each parental species (F_1_ × *M. benetos N* = 20, F_1_ × *M. zebra N* = 42). A total of 702 individuals were used in this study (Table 1). Pictures were taken with a Canon Eos 540d under standardized conditions including a ruler as length standard. All specimens were sexed, and the standard length was measured.

**Table 1:** Sampling list. The number of sampled males and females, the total number of individuals used in the study, and the rearing environment for each group

Species	Environment	Number of males	Number of females	Total
*M. benetos*	Field	32	12	44
*M. zebra*	Field	33	5	38
*M. benetos*	Laboratory	38	17	55
*M. zebra*	Laboratory	57	24	81
F1	Laboratory	49	47	96
F2	Laboratory	117	209	326
Backcross to *M. zebra*	Laboratory	22	20	42
Backcross to *M. benetos*	Laboratory	15	5	20
Total		363	339	702

### 2.3 Morphometric analyses

**Figure 1:**
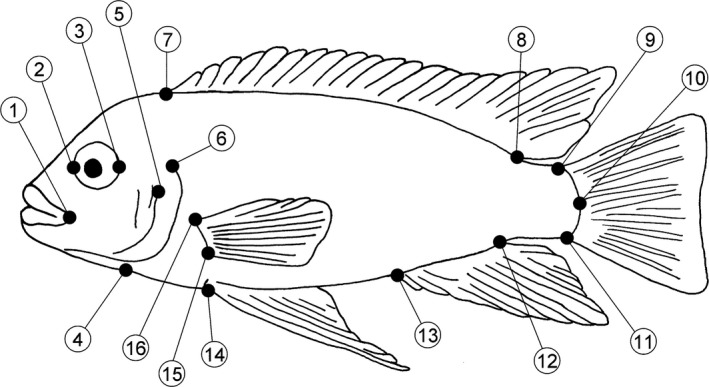
The 16 landmarks analyzed in this study: (1) most posterior point of the lips, (2) anterior edge of the eye, (3) posterior edge of the eye, (4) ventral tip of cleithrum, (5) dorsal end of pre‐opercular groove, (6) dorsal origin of operculum, (7) anterior insertion of dorsal fin, (8) posterior insertion of dorsal fin, (9) upper insertion of caudal fin, (10) midpoint of the origin of caudal fin, (11) lower insertion of caudal fin, (12) posterior insertion of anal fin, (13) anterior insertion of anal fin, (14) anterior insertion of pelvic fin, (15) ventral insertion of pectoral fin, and (16) dorsal insertion of pelvic fin

We quantified body shape variation in the two species and hybrid generations using geometric morphometric analyses. Lateral pictures of individual fish were imported into tpsDig v.2.16 (Rohlf, 2006), and we digitized 16 landmarks (Figure 1, see figure caption for a description of the landmarks). To address our question regarding the genetic and environmental components of body shape, we used data collected from wild‐caught and laboratory‐reared *M. benetos* and *M. zebra*. In a second analysis, we tried to determine the mode of gene action and test for transgressive segregation in the hybrid generations. For this, we included all laboratory‐reared parental generations and the F_1_, F_2_, and backcrosses. For each data set, landmark coordinates were aligned using least‐square superimposition as implemented in the program tpsRelw (Rohlf, 2007) to remove effects of translation, rotation, and scaling. Unless otherwise stated, all statistical analyses were performed using SPSS 20 (IBM Inc.).

### 2.4 Estimating genetic and plastic components

To distinguish genetic and plastic components of shape differentiation, we analyzed the similarities and differences of body shape in wild‐caught and laboratory‐reared populations of *M. zebra* and *M. benetos* from Mazinzi Reef. A total of 218 individuals were included in this analysis. Based on the aligned landmark coordinates, we generated a weight matrix by calculating partial warp scores with uniform components for each individual. To reduce data dimensionality, we subjected the weight matrix to a principal component analysis based on the covariance matrix of all landmarks to generate a relative warp matrix. This matrix was used as dependent variable in a multivariate analysis of covariance (MANCOVA). Species, rearing environment (wild‐caught vs. laboratory‐reared), and sex were included as factors, and standard length was used as a covariate to control for multivariate allometry. To visualize body shape variation between species and rearing environments, we calculated the divergence vector score (sensu Langerhans, 2009) of each individual fish for the first principle component of the among‐group covariance matrix for the corresponding term in the MANCOVA (Klingenberg & Spence, 1993). This allows for the visualization of body shape variation in response to a particular factor, while correcting for all other effects in the model. Individual divergence vector scores were then used as independent variables in tpsRegression (Rohlf, 2005) to generate thin‐plate spline deformation grids highlighting shape differences among groups.

### 2.5 Analyses of body shape in laboratory crosses

To investigate the mode of gene action and test for transgressive segregation in body shape, we examined the laboratory‐reared parental species and their F_1_, F_2_, and backcross hybrids for a total of 620 individuals. We calculated divergence vector scores from the species term of a MANCOVA described above and plotted the means and variances for each generation. If the body shape variation between the parental species is heritable and the genetic basis for these differences is additive, all hybrid generations' mean phenotypes are expected to be intermediate to the parentals. However, the variance in the F_2_ generation is expected to be higher in comparison with the parentals and the F_1_ hybrid generation (Lynch & Walsh, 1998). To test for additive effects, we compared the additive model of gene action to the additive‐dominance model using the joint‐scaling test to determine which model better fits our data (Lynch & Walsh, 1998). The joint‐scaling method can also be used to detect the action of epistasis; however, testing for epistasis would require more than the six lines available from our cross (P_1_, P_2_, F_1_, F_2_, BC_1_, and BC_2_). Therefore, we used a t‐test based on P_1_, P_2_, F_1_, and F_2_ data as suggested by Lynch and Walsh (1998) to evaluate epistasis.

To estimate and quantify the amount of transgressive segregation found in our crosses, we used two separate approaches. First, we employed the method developed by Stelkens et al. (2009): We removed variation due to sex and size by using the residuals from a preparatory MANCOVA, performed a PC analysis, and determined the range for the combined parentals and for the complete data set for each PC axis. The amount of transgression occurring along each axis was then calculated by subtracting the range of the parentals from the total range of the data set along the axis. The difference between the parental range and the total range was then divided by the range of the parentals. The total amount of transgression occurring in the hybrid generations (F_1_, F_2_, and backcrosses) was then calculated by summing up the transgression found on each axis adjusted for the percent of variance explained by the axis (Stelkens et al., 2009).

To parallel Stelkens et al.'s (2009) study of transgressive segregation and genetic distance in cichlids, we calculated the genetic p‐distance for the two species using 163 mitochondrial D‐Loop sequences from both species [*M. benetos N* = 85 (KC208850–KC208878, KC960378–KC960406, KC960198–KC960224), *M. zebra N* = 78 (KC208879–KC208904, KC960407–KC960434, KC960225–KC960249)] provided from previous studies (Husemann et al., 2014; Husemann *et al*., 2015). Genetic distances were calculated in MEGA5 (Tamura et al., 2011). The genetic data do not provide any information on the genetic basis of transgression or the trait, but solely is used as a measure of genetic divergence between the species pair with the intention to compare the data to the results of previous studies in other cichlids pairs from other radiations.

As sample sizes differed across generations, we performed a second analysis testing for transgression while adjusting for different sample sizes. We generated estimates of convex hull volumes for each parental species, the combined parentals, and the F_2_ generation. The convex hull of a set of points is a geometric measure describing the volume of the smallest convex set of points that contains all points in that data set. We removed variation due to sex and size by using the residuals from a preparatory MANCOVA and performed a PC analysis using the first nine axes (all axes with an Eigenvalue larger than the mean Eigenvalue) to calculate a convex hull for each group using the Quickhull algorithm (Barber, Dobkin, & Huhdanpaa, 1996). Due to different sample sizes among groups, we used a randomization procedure to calculate morphospace as described in Tobler and Carson (2010). Random distributions of morphospace were generated using 1,000 iterations of randomly selected specimens with replacement from the respective pool of individuals. A convex hull was calculated for each sample. Means and confidence intervals were calculated for each group through the examination of 1,000 iterations of this process. If body size exhibits transgressive segregation, the F_2_ is expected to occupy significantly more morphospace than that of the combined parental generations.

Finally, we also visualized body shape variation of parentals and hybrids in order to identify the nature of shape differences among groups. To do so, we generated two distinct divergence vectors: (1) We calculated a divergence vector describing the morphological gradient between the two parental species. To do so, we conducted a preparatory MANCOVA including the laboratory‐reared individuals of both parental species (sex and species served as factors, standard length as a covariate). We then calculated scores for both parental and hybrid individuals based on the first principle component of the among‐species covariance matrix. Hence, this analysis classified hybrid individuals along a morphological axis from *M. benetos*‐like to *M. zebra*‐like. F_1_ and F_2_ individuals—on average—are expected to exhibit intermediate divergence vector scores, while backcrosses should be closer to the respective parental species. (2) We calculated a divergence vector describing the morphological gradient between the combined parents (*M. benetos* plus *M. zebra*) and hybrids (F1, F2, plus backcrosses) using the same approach. This axis in part describes the effects of transgressive segregation, where hybrid individuals with a divergence score similar to that of the combined parentals exhibit no or low transgressive segregation, and increasing differences of individual divergence scores beyond the average parental score indicate increasingly transgressive phenotypes. It is important to note that this approach does not capture the entirety of transgression in multidimensional space, but it rather describes the main body shape differences between parentals and hybrids.

## 3 Results

### 3.1 Genetic and plastic components of body shape

**Figure 2:**
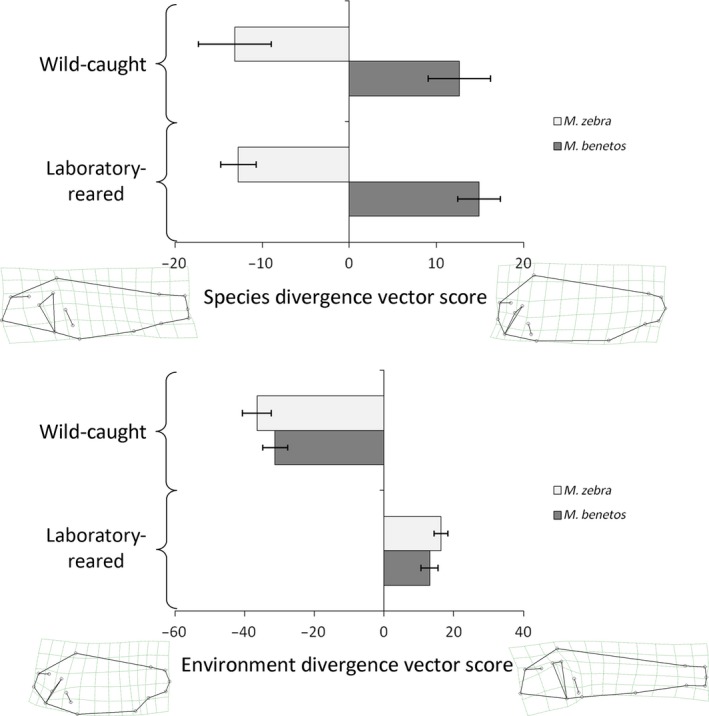
Visualization of species differences (top) and environmental effects (bottom) on body shape of *Maylandia benetos* (dark bars) and *M. zebra* (light bars) sampled in the field and raised in the laboratory. Presented are estimated marginal means (and standard errors) of divergence vector scores calculated based on the respective terms in the MANCOVA presented in Table 2

The analysis of wild‐caught and laboratory‐reared fish revealed significant plastic and genetic components that influence body shape. The greatest differences between the species, independent of the environment, were found in the shape and slope of the head and body depth (Figure 2, upper panel). When examining the differences between the treatments, wild‐caught individuals of both species had much deeper bodies with higher caudal peduncles, whereas body shape was more elongated and fusiform in the laboratory‐reared individuals (Figure 2, lower panel). The variance in the species score was higher in the wild‐caught samples compared to the laboratory‐raised fish. The MANCOVA showed that all main effects and most interaction terms were significant (Table 2); however, the effects of standard length and sex and the interaction terms involving sex were generally weak ($\eta_{p}^{2}$ < 0.15). The rearing environment (laboratory vs. field, plastic component) had the strongest effect on body shape ($\eta_{p}^{2}$ = 0.585), followed by the species identity (genetic component, $\eta_{p}^{2}$ = 0.400). The interaction of species by environment was significant as well, yet had a weaker effect ($\eta_{p}^{2}$ = 0.356).

**Table 2:** Results of the multivariate analysis of covariance (MANCOVA) of body shape in the laboratory—field comparison of *Maylandia benetos* and *M. zebra*. F‐ratios were approximated using Wilks' lambda; effect sizes were estimated with partial Eta squared ($\eta_{p}^{2}$). Significant values are printed in bold

Effect	Wilks' lambda	*F*	Hyp. *df*	Error *df*	*p*	$\eta_{p}^{2}$	Relative variance
Standard length	0.822	4.872	9.000	202.000	**<.001**	0.178	0.304
Environment	0.415	31.671	9.000	202.000	**<.001**	0.585	1.000
Sex	0.858	3.713	9.000	202.000	**<.001**	0.142	0.243
Species	0.600	14.990	9.000	202.000	**<.001**	0.400	0.684
Environment × Species	0.644	12.409	9.000	202.000	**<.001**	0.356	0.609
Sex × Species	0.930	1.694	9.000	202.000	.092	0.070	0.120
Environment × Sex	0.877	3.162	9.000	202.000	**.001**	0.123	0.210

### 3.2 Body shape in laboratory crosses

**Figure 3:**
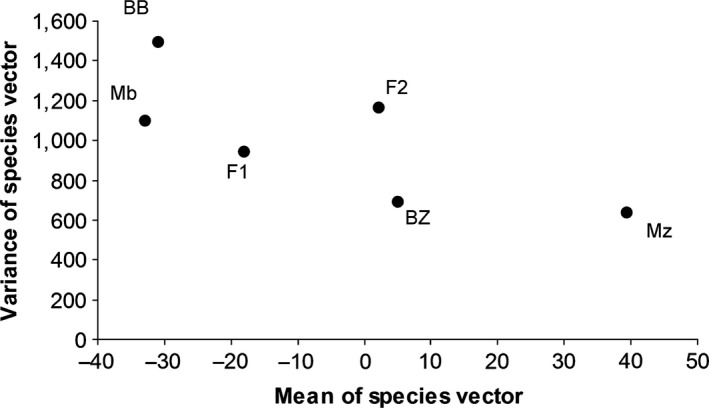
Mean vs. variance of the species vector plotted for the parental and hybrid generations

In the laboratory‐bred stocks, the means of the divergence vector scores of the parental species defined the two phenotypic extremes and the means of all hybrid generations had intermediate values relative to the parentals (Figure 3). The F_1_ divergence score mean was strongly skewed toward *M. benetos*, whereas the F_2_ mean divergence score was roughly intermediate between both parentals. The backcrosses had intermediate divergence scores, although the backcross to *M. benetos* was highly skewed toward *M. benetos*.

The analysis of the mode of gene action of body shape differences via the joint‐scaling test rejected additivity (*p*.A = 0). The rejection of the additive model was supported by the plot of means and variances of each generation (Figure 3). If the phenotype follows the additive genetic model, a plot of means and variances for each generation would produce a triangular pattern with the parental points defining the base of the triangle and the F_2_ its apex (Barson, Knight, & Turner, 2007). Our data clearly deviate from these expectations: While the parentals represented the morphological extremes (*M. benetos N* = 55, μ = −32.81, σ^2^ = 1094.27; *M. zebra N* = 81, μ = 39.45, σ^2^ = 637.02), the backcross to *M. benetos* had the highest variance (*N* = 20, μ = −30.83, σ^2^ = 1493.08). Further the F_1_ (*N* = 96, μ = −18.06, σ^2^ = 943.7731) and both backcrosses deviated from the expected values under additivity and were skewed toward *M. benetos*. In addition, the variance of the F_1_ generation was lower than that observed in *M. benetos*. The variance of the F_2_ (*N* = 326, μ = 2.28, σ^2^ = 1164.61) was only slightly higher than that in *M. benetos* (Figure 3). The additive‐dominance model was rejected as well (*p*.AD = 1.01 × e^−14^); yet, when comparing both models, the additive‐dominant model explains the data slightly better (*p*.A.AD = 4.68 × e^−08^). As we did not have sufficient hybrid lines, we could not use the joint‐scaling test for epistasis. Instead we used the test based on the variances of the parental lines, F_1_ and F_2_ as proposed by Lynch and Walsh (1998). The *t*‐test could not reject epistatic effects (test statistic: −0.22; epistasis can only be rejected if this value is above 1.96; Lynch & Walsh, 1998).

**Figure 4:**
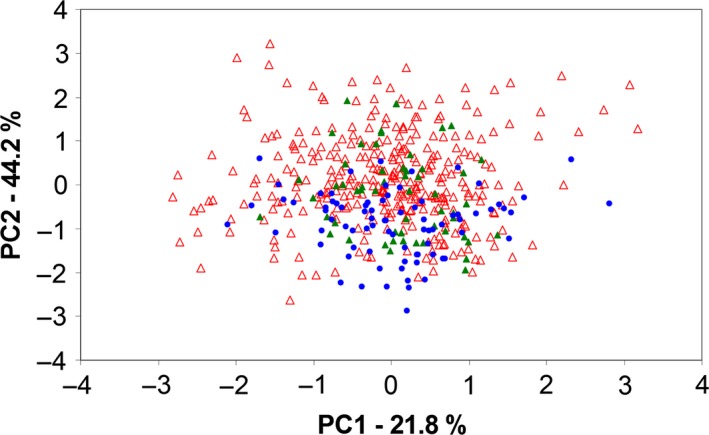
Transgressive segregation in body shape. PC1 and PC2 for body shape were plotted for the *Maylandia benetos* (green triangles), *M. zebra* (blue kites), and F_2_ hybrids (red triangles). Note that only the first two PC axes are shown, and therefore, the total amount of transgressive segregation is not displayed here

Using the approach provided by Stelkens et al. (2009), our data show clear signs of transgressive segregation (Figure 4, Table 3). The range of the F_2_ phenotypes clearly exceeds the ranges of shape space on each PC of the combined parentals (Figure 4). The amount of transgression found in the combined hybrid generations differed between 7% (PC4) and 52.1% (PC3) (Table 3). The total amount of transgression across all axes and adjusted for the variance explained by each axis was 23.8% (Table 3).

**Table 3:** Amount of transgressive segregation found at each axis calculated according to Stelkens et al. (2009). Recorded is the amount of transgression expressed as a % found at each PC axis (TSPCi). The total amount for transgression across all axes and adjusted for the variance explained by the axis was 23.8 %

	PC1	PC2	PC3	PC4	PC5	PC6	PC7	PC8	PC9
% explained	20.91	15.66	9.53	6.84	6.04	4.98	4.45	3.89	3.6
Parentals range	4.9224	4.2328	3.8416	5.7858	4.7983	4.5127	5.7604	4.5817	4.5934
Total range	5.9971	6.10415	5.8426	6.1883	5.3630	6.7750	6.2585	6.2241	6.9664
TSPCi (%)	21.8	44.2	52.1	7	11.8	50.1	8.6	35.8	51.7

Within the 85 sequences of *M. benetos,* we found five haplotypes with a haplotype diversity of 0.278 and a nucleotide diversity of 0.0005; the 78 sequences for *M. zebra* contained nine haplotypes with a haplotype diversity of 0.624 and a nucleotide diversity of 0.0023. The genetic *p*‐distance between the two taxa calculated from 163 D‐Loop sequences was 0.002 (SE 0.004). The within‐group distances within *M. zebra* and *M. benetos* were 0.002 and 0.000, respectively.

**Figure 5:**
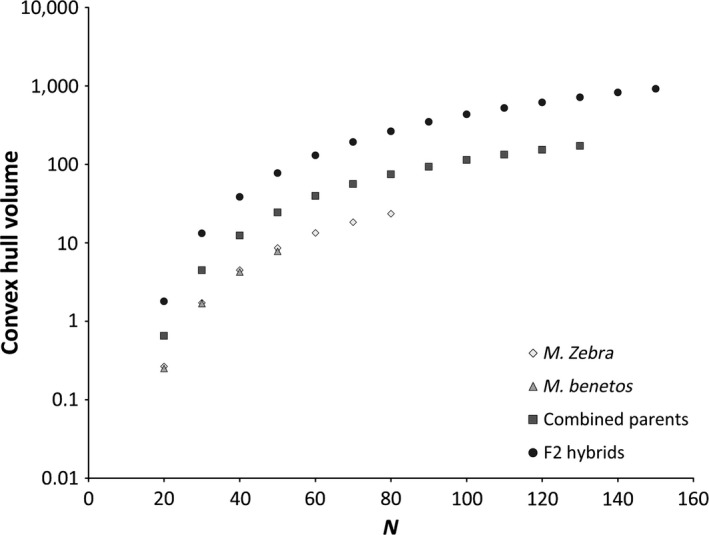
Comparison of morphospace occupation (Convex hull volume) in each of the parental species, both parentals combined, and the F_2_ hybrid generation adjusted to different sample size using the Quickhull algorithm; 95% confidence intervals are too narrow to be visible (see online supplement Table for actual values)

**Figure 6:**
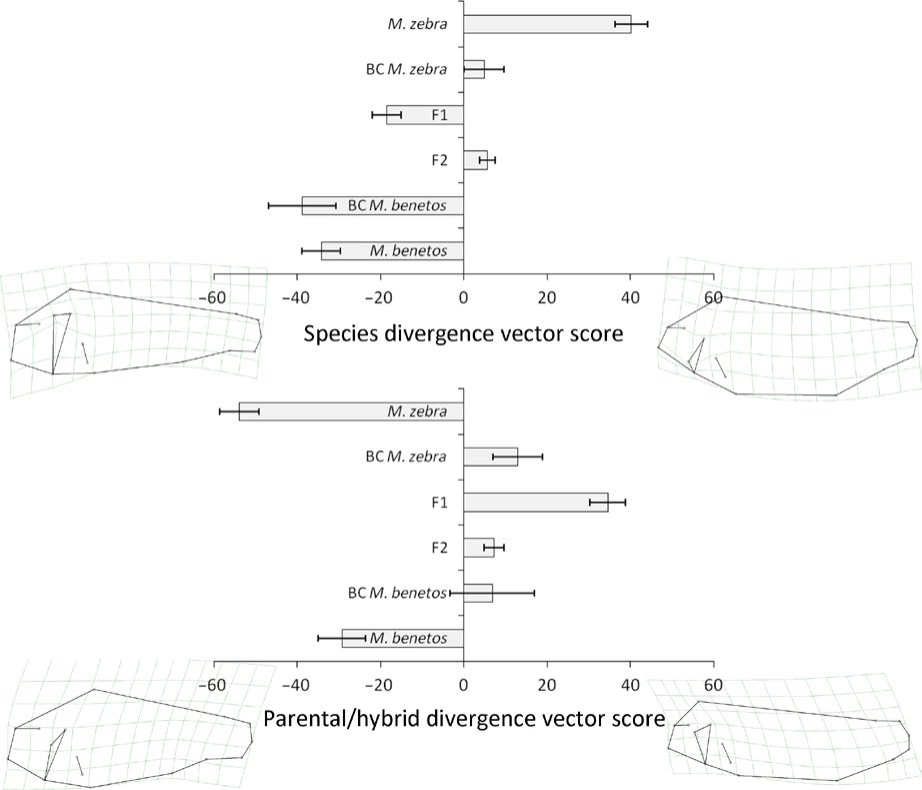
Visualization of the nature of transgressive phenotypes: species and generational differences along the species divergence vector (top) and along the parental/hybrid divergence vector (bottom). Presented are estimated marginal means (and standard errors) of divergence vector scores

The convex hull analysis confirmed our finding of transgressive segregation when adjusting for sample size (Figure 5). All 95% confidence intervals were extremely small and did not overlap. The two parental species are fairly similar in morphospace occupation. The F_2_ generation has a higher convex hull volume than the combined volume of the parental species independent of the sample size. Thus, the parental species occupy only a subset of the overall morphospace occupied by hybrids. The nature of transgressive segregation is further visualized in Figure 6. We show that the intermediate hybrid individuals generally have shallower bodies than the parentals and have a more fusiform shape (Figure 6).

## 4 Discussion

In this study, we performed a common garden experiment and hybrid crosses to estimate the potential of plasticity and transgressive segregation contributing to body shape variation in a sympatric pair of closely related rock‐dwelling cichlids. We found that the body shape differences between the two species were largely maintained despite clear differences between wild‐caught and common garden‐raised fish. The mode of gene action underlying variation in body shape is complex, and the additive and additive‐dominance models were both rejected. Furthermore, the analysis of body shape variation in hybrids revealed a high potential for transgressive segregation despite low genetic divergence between the species suggesting that transgression as well as plasticity may generate considerable amount of phenotypic variation. Transgressive phenotypes resemble some other taxa within the Lake Malawi cichlid radiation suggesting that some of the radiation may have been seeded by variation resulting from hybridization.

### 4.1 Genetic and plastic components of body shape

Our data clearly show that species differences in body shape have a genetic basis. Despite significant differences between wild‐caught and laboratory‐reared fish, body shape differences observed between the two species in the wild are maintained after ~12 generations in a laboratory environment (Figure 2). In addition, the data from hybrids support a genetic component of body shape with the F_1_ and F_2_ generation being roughly intermediate between the two parental species (Figures 3 and 6). This strong genetic component to variation body shape suggests that the species are adapted to different micro‐niches in nature and that this adaptation is the result of habitat‐ or community‐specific selective pressures (Husemann et al., 2014).

Nonetheless, there is also a strong plastic component to body shape: Both species consistently changed body shape under standardized laboratory conditions (Figure 2). The changes followed a similar path, as both species became more slender with shallower bodies and thinner caudal peduncles. This might be a plastic response to smaller spaces, less activity, a structurally less complex habitat, and/or reduced predation pressure in the laboratory (Kerschbaumer et al., 2011). The observed plasticity in body shape may play an adaptive role in the diversification of Lake Malawi cichlids; this plasticity may allow species to rapidly respond to novel environmental challenges. This in turn may prevent competitive exclusion in species‐rich and highly competitive communities like those observed in rock‐dwelling cichlids (Ding, Daugherty et al., 2014; Ghalambor et al., 2007; Olsson & Eklöv, 2005). Furthermore, a plastic response can lead to heritable adaptive changes, if selection favors a specific character state within the reaction norm across multiple generations (Via et al., 1995, see below).

Plasticity in body shape is not surprising as cichlids exhibit plasticity in a variety of morphological traits, such as jaw morphology and dentition (Muschick, Barluenga, Salzburger, & Meyer, 2011), body shape (Wimberger, 1992), mouth orientation, the size and orientation of fins, and the thickness of the caudal peduncle (Kerschbaumer et al., 2011). Interestingly, we find many similar traits to be plastic in our hybrid crosses: Body depth, orientation of the mouth, and the thickness of the caudal peduncle show strong variation between environments (Figure 2). These traits are important for feeding and swimming performance, and therefore, they can be assumed to be under strong ecological selection (Langerhans & Reznick, 2009). However, even in traits under strong selection, high genetic variability can be maintained if strong species‐by‐environment interactions occur (Greenfield, Danka, Gleason, Harris, & Zhou, 2012). Such interactions, similar to genotype‐by‐environment interactions, may provide the genetic variation to quickly react to environmental change via plastic responses (Rodriguez, 2012). The resulting phenotypic plasticity may have contributed to the large phenotypic diversity observed in the East African cichlid radiation.

### 4.2 Analyses of hybrid crosses

In a second step, we hybridized our two model species to get some insights into the genetic basis of body shape in these species. Little is known about the genetic basis of fish body shape. In sticklebacks, divergence in body shape between marine and freshwater lines appears to be determined by many genes (Schluter et al., 2004). In turn, a QTL analysis of the same stickleback species indicated that body shape is determined by few genes with large effects in addition to multiple genes with smaller effects (Albert et al., 2007). In cichlids, the genetic basis of body shape has been investigated in a pair of crater lake cichlids (Franchini et al., 2014). The study used genomewide SNPs and geometrics morphometrics to identify QTL and found that few genetic regions of large effects contribute to the divergence along the benthic‐limnetic axis (Franchini et al., 2014). However, several other traits, including tropic morphology, coloration, and mate choice, have been studied (Albertson & Kocher, 2005, 2006; Albertson, Streelman, & Kocher, 2003a,b; Ding, Curole et al., 2014; O'Quin, Drilea, Conte, & Kocher, 2013; O'Quin, Drilea, Roberts, & Kocher, 2012): These studies, for example, suggested that one to eleven genetic factors underlie shape differences of individual elements of trophic structures, and pleiotropic effects appear to be a common feature in the genetic architecture of these traits (Albertson et al., 2003a). Our data similarly indicate a complex genetic basis of body shape. The additive and dominant modes of gene action were rejected and epistatic interactions seem likely. Despite our limited knowledge, our data and previously published studies suggest that body shape represents a composite trait (see also Selz et al., 2014) with a complicated genetic architecture that does not follow a simple additive model. However, we have to acknowledge that sample sizes in this study are relatively low for quantitative genetic analyses, and hence, no further conclusion can be drawn. Future quantitative genetic and developmental studies are required to understand the genetic basis of body shape in cichlids more comprehensively.

### 4.3 Transgressive segregation

Another source of phenotypic variation is transgressive segregation resulting from hybridization of distinct genetic lineages. Hybridization is often considered a force leading to a decline of biodiversity, because it disrupts species boundaries (e.g., Perry, Lodge, & Feder, 2002). However, the elevated genetic and phenotypic variance resulting from hybridization provides new variation that selection can act upon. In this way, hybridization and the resulting transgressive segregation can lead to the evolution of new adaptive phenotypes (e.g., Genner & Turner, 2012; Rieseberg, Raymond et al., 2003; Seehausen, 2004).

A variety of cichlid phenotypes are known to exhibit transgressive segregation. Albertson and Kocher (2005), for example, have shown that the cichlid skull is susceptible to transgressive segregation, which is in line with our findings, although transgressive segregation in the jaw seems limited by the genetic architecture of this phenotype. Parsons, Son, and Albertson (2011) confirmed high degrees of transgressive segregation in head shape in a pair of Malawi rock‐dwellers. Further, transgressive segregation has been detected for a coloration phenotype in Lake Malawi rock‐dwellers (O'Quin et al., 2012). This high potential for transgressive segregation in different phenotypes suggests that hybridization can promote evolvability in East African cichlids and might be an important mechanism in generating new variation for selection to act on (Parsons et al., 2011; Seehausen, 2004; Stelkens et al., 2009). This has led some to suggest that transgressive segregation resulting from hybridizations at a variety of taxonomic scales has contributed to the origin of new species (Smith, Konings, & Kornfield, 2003), genera (Albertson & Kocher, 2005), and even whole clades (Genner & Turner, 2012) of Lake Malawi cichlids.

**Figure 7:**
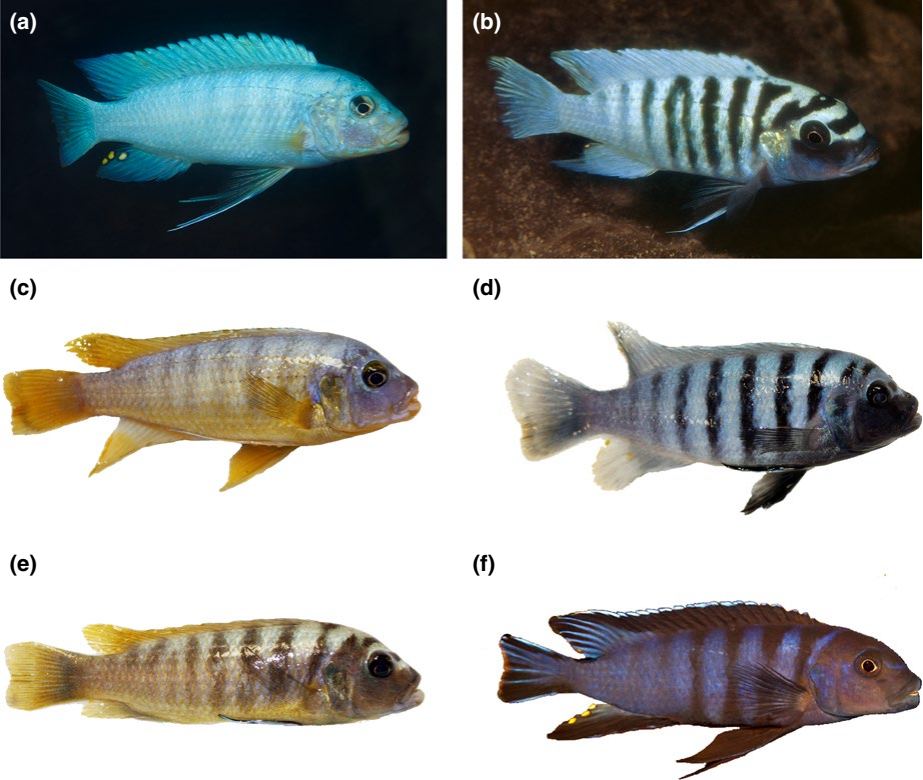
Pictures of males of (a) *Maylandia benetos* and (b) *M. zebra* from the field (Mazinzi Reef, picture credit A. Konings) and the laboratory (c, d), and of an (e) F_2_ hybrid between the two species with a transgressive phenotype; (f) individual of *Pseudotropheus* “elongatus yellowtail” from Mumbo Island (picture credit A. Konings) with a similar elongated body shape

Our findings suggest that body shape is yet another cichlid phenotype exhibiting transgressive segregation: Hybrids occupy body shape morphospace beyond what is found in the parentals indicating significant transgression. F_1_ and F_2_ hybrids have more fusiform bodies in comparison with both parentals, a phenotype resembling the “aggressive” and “elongata” species groups within the genus *Pseudotropheus* (Figure 7; Konings, 2007). The amount of transgression found in the hybrid crosses was 23.8 %, which is remarkably high for a cross of two species so closely related (Stelkens & Seehausen, 2009; Stelkens et al., 2009). It has to be noted, however, that the approach for quantification is dependent on the coordinate system used. New less biased approaches need to be developed to make results more comparable across studies. The genetic distance between these two *Maylandia* species is estimated at 0.002 (based on mitochondrial D‐Loop sequences), which is lower than any of the distances separating other cichlid hybrids reporting transgressive segregation (Albertson & Kocher, 2005; Genner & Turner, 2012; Stelkens et al., 2009). Furthermore, the amount of transgression observed in our study exceeded estimates of transgression for F_2_ generations of taxa with an order of magnitude higher genetic divergence. This runs counter to the typical pattern of transgressive segregation in which the degree of phenotypic novelty increases with genetic distance and suggests that hybridization can lead to high amounts of transgression, even in very closely related species such as those commonly found in Lake Malawi. In addition, the observation of transgressive segregation in two such closely related species suggests that, even within very recently diverged Lake Malawi cichlids, stabilizing selection on body shape can play an important role in shaping the observed phenotypic diversity in the lake (Rieseberg et al., 1999). Overall, our results and those of previously published studies suggest that transgressive segregation seem to be the rule rather than the exception in cichlids, and as such, transgressive segregation may be an important mechanism in generating phenotypic variation in cichlids and may have played an important role in the evolution of this adaptive radiation.

## 5 Conclusions

Our analysis of body shape in wild‐caught and laboratory‐reared specimens of two closely related species of cichlids revealed that species‐specific differences have a genetic basis. In addition, body shape has a plastic component providing the potential to promote and maintain diversity. The mode of gene action of the species differences is complex, likely polygenic, and involves dominant and epistatic interactions. The potential for transgressive segregation is high, supporting the possibility of an important role of hybridization of closely related species in cichlid diversification. Therefore, our study supports the idea that plasticity and transgressive segregation as result of occasional hybridization may have been important factors in the evolution of the Lake Malawi cichlid radiation and potentially in other rapidly diverging systems.

## Conflict of Interest

None declared.
