# Supplementary material for: Body shape differences in a pair of closely related Malawi cichlids and their hybrids: Effects of genetic variation, phenotypic plasticity, and transgressive segregation
Source: Ecol Evol. 2017 May 10;7(12):4336–46. doi: 10.1002/ece3.2823 (PMC5478046; doi:10.1002/ece3.2823)
Supplement: Supplementary file 1 [file ECE3-7-4336-s001.doc]

**Supplementary table 1. Results from Convex Hull calculations for different sample sizes.**

| N | F2 | CI | Combined parents | CI | *M. benetos* | CI | *M. zebra* | CI |
| --- | --- | --- | --- | --- | --- | --- | --- | --- |
| 20 | 1.79554437 | 0.08351757 | 0.650838639 | 0.031439089 | 0.250778604 | 0.013666126 | 0.265479907 | 0.01288 |
| 30 | 13.15862251 | 0.432280453 | 4.480043283 | 0.149693563 | 1.678837558 | 0.066002032 | 1.715140539 | 0.05624 |
| 40 | 38.38659836 | 1.022714081 | 12.39341453 | 0.340800968 | 4.244189956 | 0.132226864 | 4.517667015 | 0.12667 |
| 50 | 77.58670015 | 1.890211296 | 24.32731279 | 0.601825284 | 7.783068019 | 0.204898221 | 8.635567027 | 0.19617 |
| 60 | 129.8158609 | 2.893708136 | 39.47963463 | 0.825430516 |  |  | 13.36519128 | 0.27419 |
| 70 | 192.9804036 | 3.933205831 | 55.97610597 | 1.074580568 |  |  | 18.33477137 | 0.33447 |
| 80 | 263.8608326 | 4.683649789 | 74.7302328 | 1.296784238 |  |  | 23.48108492 | 0.37378 |
| 90 | 347.5564014 | 6.137878858 | 93.06349392 | 1.502359975 |  |  |  |  |
| 100 | 434.3340893 | 6.889124269 | 113.3173337 | 1.744341356 |  |  |  |  |
| 110 | 523.3316976 | 7.915917659 | 133.2686942 | 1.838658441 |  |  |  |  |
| 120 | 613.7438576 | 9.63999866 | 153.0135193 | 2.008608548 |  |  |  |  |
| 130 | 716.534681 | 10.49160869 | 171.4985345 | 2.114248723 |  |  |  |  |
| 140 | 823.3080613 | 11.09467135 |  |  |  |  |  |  |
| 150 | 917.2326636 | 11.92444308 |  |  |  |  |  |  |
